# Supplementary material for: Comparative Study of Short-Term Efficacy and Safety of Mitomycin versus Lobaplatin for Hyperthermic Intraperitoneal Chemotherapy after Radical Surgery in Colorectal Cancer with High-Risk Factors for Peritoneal Carcinomatosis: A Propensity Score Matching Analysis
Source: Curr Oncol. 2023 Jan 21;30(2):1488–501. doi: 10.3390/curroncol30020114 (PMC9955354; doi:10.3390/curroncol30020114)
Supplement: Supplementary file 1 [file curroncol-30-00114-s001.zip › curroncol-2121576-supplementary.pdf]

Supplementary Table S1 Definition of CTCAE V5.0 adverse events

adverse event	definition	I	II	III	IV
Anemia	A disease characterized by a decrease in the total amount of hemoglobin in 100 ml of blood	Hemoglobin < lower limit of normal value ~ 10.0 g / dl<Lower limit of normal value ~ 6.2mmol/l<Lower limit of normal value ~ 100g / L	Hemoglobin < 10.0 ~ 8.0g/dl<6.2 ～ 4.9mmol/L；< 100～ 80g/L	Hemoglobin < 8.0g/dl<4.9mmol/L；< 80 g/L；Blood transfusion is needed	Life threatening; Urgent treatment is needed
Hypoalbuminemia	The results of laboratory examination showed that the concentration of albumin in blood was low	< lower limit of normal value ~ 3 g / dl<Lower limit of normal value ~ 30 g / L	<3～2 g/dL；<30～20 g/L	<2 g/dL；<20 g/L	Life threatening; Urgent treatment is needed
Myelosuppression	A disease characterized by decreased hematopoietic function of bone marrow	Slightly too few cells or a decrease of ≤ 25% compared with the total number of normal cells in this age group	Moderately too few cells or a decrease of > 25% and < 50% compared with the total number of normal cells in this age group	Severe cytopenia or reduction of > 50% and ≤ 75% compared with the total number of normal cells in this age group	The aplastic disorder lasted for more than 2 weeks
Wound complications	New abnormalities in the existing wound site	It can be observed that local treatment is needed	Local care is required	Surgery is needed	Life threatening; Urgent treatment is needed

Abdominal infection	Abdominal infection	-	Oral administration is required (e.g. antibiotics, antifungal or antiviral drugs)	Intravenous antibiotics, antifungal or antiviral drugs are needed; Invasive treatment is needed	Life threatening; Urgent treatment is needed
Pulmonary infection	Infectious diseases, including pneumonia, occurring in the lungs.	-	Moderate symptoms; Oral medication (antibiotics, antifungal or antiviral drugs) is required	Intravenous antibiotics, antifungal or antiviral drugs are needed; Invasive treatment is needed	Life threatening; Urgent treatment is needed
Postoperative bleeding	Postoperative disorder characterized by bleeding	The symptoms are mild and need no treatment	Moderate bleeding requiring blood transfusion (blood transfusion volume < 2 units (infants < 10cc / kg))	Blood transfusion volume > 2 units (infants > 10 cc / kg), invasive treatment and hospitalization are required	Life threatening; Urgent treatment is needed
Anastomotic leakage	Rupture of gastrointestinal anastomosis and leakage of contents	The diagnostic findings were asymptomatic; No treatment required	Symptomatic; Need treatment	Serious; Invasive treatment is needed	Life threatening; Urgent treatment is needed
Ileus	A disease characterized by the inability of the gut to transport intestinal contents	No clinical symptoms, only radiation observation is required	Symptomatic; Gastrointestinal function changes; fasting	Severe changes of gastrointestinal function; Total parenteral nutrition is required; Catheterization is needed	Life threatening; Urgent treatment is needed
